# Fault Detection of Planetary Gears Based on Signal Space Constellations

**DOI:** 10.3390/s22010366

**Published:** 2022-01-04

**Authors:** Maite Martincorena-Arraiza, Carlos A. De La Cruz Blas, Antonio Lopez-Martin, Cristián Molina Vicuña, Ignacio R. Matías

**Affiliations:** 1 Institute of Smart Cities, Public University of Navarre (UPNA), Campus Arrosadia, 31006 Pamplona, Spain; maite.martincorena@unavarra.es (M.M.-A.); carlos.aristoteles@unavarra.es (C.A.D.L.C.B.); natxo@unavarra.es (I.R.M.); 2 Laboratorio de Vibraciones Mecánicas, Departamento de Ingeniería Mecánica, Universidad de Concepcion, Edmundo Larenas 219, Concepcion 4070409, Chile; crimolin@udec.cl

**Keywords:** planetary gearbox, vibration signal processing, fault detection

## Abstract

A new method to process the vibration signal acquired by an accelerometer placed in a planetary gearbox housing is proposed, which is useful to detect potential faults. The method is based on the phenomenological model and consists of the projection of the healthy vibration signals onto an orthonormal basis. Low pass components representation and Gram–Schmidt’s method are conveniently used to obtain such a basis. Thus, the measured signals can be represented by a set of scalars that provide information on the gear state. If these scalars are within a predefined range, then the gear can be diagnosed as correct; in the opposite case, it will require further evaluation. The method is validated using measured vibration signals obtained from a laboratory test bench.

## 1. Introduction

Planetary gear (PG) transmissions are widely used as mechanical elements in the automotive industry [1], aeronautics [2], wind power generation [3], etc. Generally, these mechanisms are responsible for using the necessary power supplied by the motors to execute the functions associated with each application, so that the detection of faults in predictive maintenance in these elements is crucial in terms of safety and cost. Research of the vibrations for fault detection in gearboxes began in the late 1970s [4]. Since then, researchers have worked on the definition of numerical indicators using, generally, experimental vibration signals obtained from PG-scale models. Initially, simple time domain parameters such as energy, amplitude, and statistical moments were taken into account to verify the presence or the absence of failures. Later, in order to improve the behavior of these indicators, signal preprocessing was introduced to enhance fault detection [5,6,7]. Subsequently, fault indicators were developed that analyze the signal behavior in the frequency domain [8,9,10] and in time–frequency domains simultaneously [10,11,12]. This way, more complete information of the vibration signal was obtained and, in the case of time–frequency techniques, its variation over time. This allows a more robust fault diagnosis and a better understanding of the temporal variability of the vibrations in a PG. However, most of these techniques are based on a single parameter and strongly related to the magnitude spectrum.

In recent years, there is a great interest in developing monitoring techniques for the predictive maintenance of rotary elements such as PGs. However, there is not always accessibility to test signals obtained from real machines, so many efforts have also been made in modeling vibration signals, developing several methods. Initially, the problem was addressed from a physical point of view through the dynamic model [13,14,15] which, using concepts such as forces, inertia, damping and stiffness of the bodies, defines the dynamics in a PG through integro-differential equations. As a complementary approach, the finite element method (FEM) [16,17,18] has also been used to approximate these complex differential equations and model the deformations of bodies in three dimensions. To achieve this, the FEM method is based on dividing a solid into a set of finite elements and defining algebraic equations for each element. This way, once the boundary conditions, initial conditions and loads are defined, solving these algebraic equations gives an approximation to the partial differential equations over the entire surface. More recently, the phenomenological model [19,20,21] was proposed as a simpler and more intuitive alternative to the dynamic model. The phenomenological model obtains the vibration signals through simpler algebraic equations based on empirical observations providing an intuitive understanding of the characteristics of the vibration signal spectrum.

In this paper, a new processing method based on the phenomenological model is proposed. The approach is based on a projection of the vibration signals onto an orthonormal basis formed from the healthy signals. Thus, the measured signals can be represented as a linear combination of the basis. If such combination is within a predefined range, the PG can be diagnosed as correct; in the opposite case, the PG will require a preventive intervention. The basis is obtained, first calculating the low pass (baseband) components of the original healthy band pass signals and, subsequently, implementing the Gram–Schmidt’s orthogonalization method.

## 2. Materials and Methods

**Figure 1:**
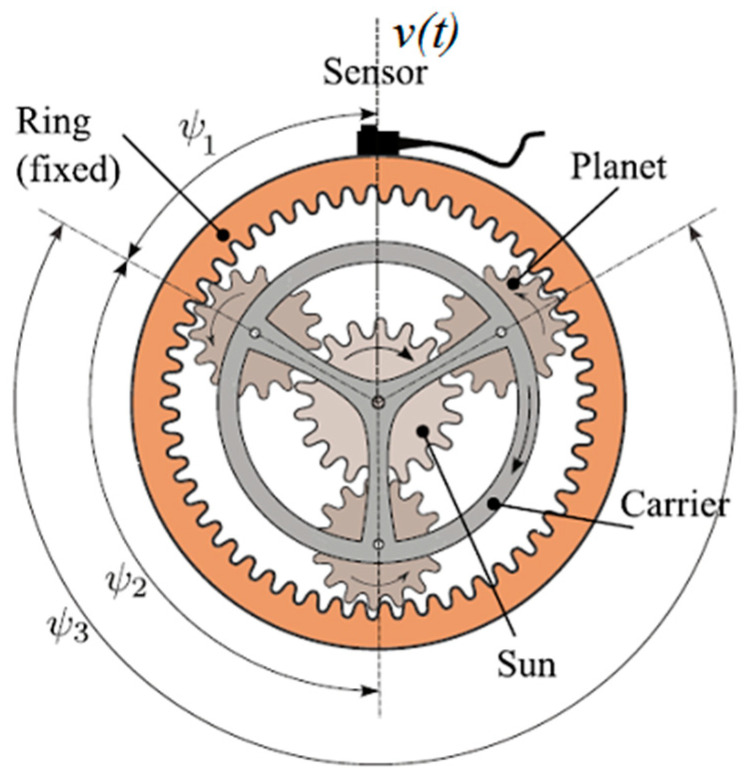
Schematic view of a PG [22].

A planetary gear set (see Figure 1) is a system consisting of one or more external gears or planets that rotate on a central gear or sun. The planets are mounted on a structure called carrier that can rotate relative to the planets and sun with an external, stationary gear called the ring so the planets mesh with them.

The main components and characteristics of a PG are listed below:

- Ring: it is fixed and contains *Z*_*R*_ teeth. Hence the rotation frequency is *f*_*R*_ = 0.
- Sun: input component (*Z*_*S*_ teeth). Rotation frequency *f*_*S*_.
- Planets: *Z*_*P*_ teeth each planet. Rotation frequency *f*_*P*_.
- Carrier: output component. Rotation frequency *f*_*C*_.

The transducer used to measure vibrations in the gearbox is an accelerometer located at a fixed position as shown in Figure 1. In this way, all the information to be analyzed (the vibration signal) is obtained from the same record. The acquired vibration signal will be the combination of vibrations produced by the simultaneous gear meshing processes at different points of the PG. In addition, each of these vibrations will travel a different path to the sensor. When the gear system is in good condition, the sensor will detect a particular and well-defined signal shape, which will be modified when a fault begins to manifest.

### 2.1. Modeling of the Vibration Signal

The phenomenological model (PM) [19,20] defines the vibration signal in a PG as the sum of the vibrations produced by the planet–sun and planet–ring gear meshing processes, modelled as periodic signals with a period equal to the gear mesh period (related to the number of teeth and the rotation period of the carrier). Each of the vibrations will travel a different path to the sensor in the ring, which generates a modulation of amplitude in the resulting vibration signal.

Figure 1 shows the PG example on which the development of the PM will be based. The PG is composed of 3 equally spaced planets, whose initial angular position is calculated by the formula $\Psi_{i}=\left(2\pi \left(i-1\right)\right)/N_{P}$, where $N_{P}$ is the number of planets and i = 1, 2,…, $N_{P}$ is the planet index.

The total vibration signal, *v(t)*, is composed by the sum of the vibration signals produced by the gear meshing process of planet *i* with the ring and the sun ($v_{i}\left(t\right)$ = $v_{ri}\left(t\right)$ + $v_{si}\left(t\right)$) as would be appreciated by an observer located on the carrier. Each of these signals is an amplitude modulated by $A_{i}\left(t\right)$, i = 1, 2 …, $N_{P}$ due to the periodically variable transmission path from the vibration sources to the sensor. That is, the amplitude of the vibration signal will vary according to the distance to which the planet is located with respect to the sensor attached to the ring.

#### 2.1.1. Transmission Paths of the Vibration Signal

In [20], the different transmission paths from the vibration source to the sensor are described. However, only two of these paths modulate the vibration signal, since the rest are time invariant and generate a constant energy loss with no crucial information. The two paths taken into account are defined as:

1 Planet–ring mesh point 🡪 ring 🡪 transducer. Defined by a modulation function $A_{ri}\left(t\right)$.
2 Planet–sun mesh point 🡪 planet 🡪 ring 🡪 transducer. Defined by a modulation function $A_{si}\left(t\right)$.

In general, the shape of function $A_{ri}\left(t\right)$ will be the same as $A_{si}\left(t\right)$ and only the amplitude will vary. This function contains the spectral information of the sidebands for a particular PG, so it is necessary that its periodicity correspond to the frequency of the carrier *f*_*C*_. There are different options to simulate the $A_{ri}\left(t\right)$ function. In [20], a raised cosine with period *f*_*C*_ is used, while in [19] a bell-shaped function with the same frequency is chosen. In both cases, the function has a certain DC component, since, in reality, while the PG is in motion, the vibration detected by the sensor will be higher or lower depending on the distance at which the source of the vibration is, but it will never be null. From these two options, in this work a raised bell function has been chosen to model the transmission paths and derive the proposed method:

(1) $$A_{r}\left(t\right)=DC_{lev}+Ae^{-2\pi f_{c}t^{2}}$$

where $DC_{lev}$ corresponds to the DC level mentioned above and A is the amplitude of the bell. The functions of the planets ($A_{ri}\left(t\right)$) can be obtained, as well as the functions corresponding to the path from the sun–planet mesh point ($A_{si}\left(t\right)$) as:

(2) $$A_{ri}\left(t\right)=A_{r}\left(t+\frac{i-1}{N_{p}}T_{c}\right)$$

(3) $$A_{si}\left(t\right)=kA_{ri}\left(t\right)$$

for *i* = 1, 2, …, *N_p_*. The transmission path defined by $A_{si}\left(t\right)$ is considered the longest one and, therefore, a greater energy loss is expected. Thus, *k* is a factor describing the energy loss (*k* < 1) [20].

#### 2.1.2. Vibration Signal

The vibration signals produced by the gear-meshing processes (see Figure 1) between each planet and the sun ($v_{spi}\left(t\right)$) and each planet and the ring $v_{rpi}\left(t\right)$ are periodic functions with frequency equal to the gear mesh frequency $w_{m}$ = $Z_{R}w_{c}$

(4) $$v_{rpi}\left(t\right)=V_{rp}\cos\left(w_{m}\left(t+\frac{\Psi_{i}+\delta_{pi}}{w_{c}}\right)\right)$$

(5) $$v_{spi}\left(t\right)=V_{sp}\cos\left(w_{m}\left(t+\frac{\Psi_{i}+\delta_{pi}}{w_{s}-w_{c}}\right)+\gamma_{i}\right)$$

where $\Psi_{i}=\frac{2\pi \left(i-1\right)}{N_{p}}$ is the angular position of each planet, *γ_i_* the phase difference between the sun–planet and the ring–planet gear-meshing processes and *δ_pi_* the angular displacement of the planet that can occur due to fabrication errors or a fault/crack in the carrier, among other causes. In this case, these type of failures will not be taken into account (i.e., *δ_pi_* = 0).

By adding the modulation due to the transmission paths and taking into account that the vibration detected in the sensor is the superposition of all the vibrations produced in the PG, the final vibration signal is:

(6) $$v\left(t\right)=\overset{N_{p}}{\underset{i=1}{\sum }}A_{ri}\left(t\right)v_{rpi}\left(t\right)+A_{si}\left(t\right)v_{spi}\left(t\right)$$

Substituting (1) to (5) in (6), it leads to:

(7) $$\begin{array}{cc} v\left(t\right)=\overset{N_{p}}{\underset{i=1}{\sum }}[DC_{lev} & +A_{r}e^{-2\pi f_{c}{\left(t-\frac{i-1}{N_{p}}T_{c}\right)}^{2}}]V_{rp}\cos\left(w_{m}\left(t-\frac{\Psi_{i}+\delta_{pi}}{w_{c}}\right)\right)\\ & +k\left[DC_{lev}+A_{r}e^{-2\pi f_{c}{\left(t-\frac{i-1}{N_{p}}T_{c}\right)}^{2}}\right]V_{sp}\cos\left(w_{m}\left(t-\frac{\Psi_{i}+\delta_{pi}}{w_{s}-w_{c}}\right)+\gamma_{i}\right)\;\; \end{array}$$

The values of $\gamma_{i}$ and $\delta_{pi}$ depend on the characteristics of the PG, which can be classified in the following groups [19]:

- Group A: PGs with equally-spaced planet gears and in-phase gear meshing processes ($\delta_{pi}=0$; $\gamma_{i}=0$).
- Group B: PGs with equally-spaced planet gears and out-of-phase meshing processes ($\delta_{pi}=0$; $\gamma_{i}\neq 0$).
- Group C: PGs with unequally-spaced planet gears and in-phase meshing processes ($\delta_{pi}\neq 0$; $\gamma_{i}=0$).
- Group D: PGs with unequally-spaced planet gears and out-of-phase meshing processes ($\delta_{pi}\neq 0$; $\gamma_{i}\neq 0$).

**Figure 2:**
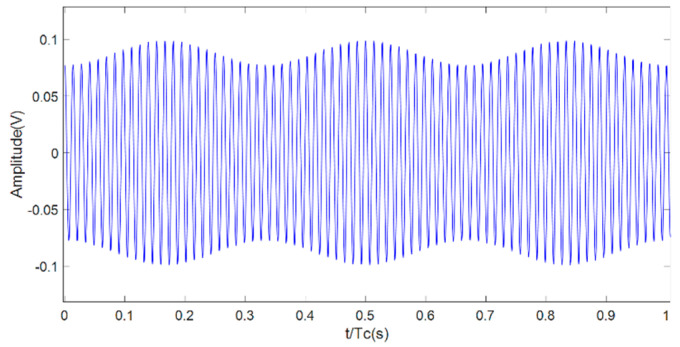
Vibration signal of a healthy PG.

Figure 2 shows as an example of the resulting modeled vibration signal of a healthy PG belonging to group A with $DC_{lev}$ = 10 mV, A = 50 mV, and unity value of $V_{rp}$ and $V_{sp}$. This group is the most common one and it will be used to demonstrate the proposed technique with experimental results.

### 2.2. Proposed Signal Processing Method

As mentioned above, the vibration signal is formed by a high frequency component, i.e., the fundamental frequency, known as gear mesh frequency, corresponding to the gear meshing processes of the different gears and a lower frequency envelope due to the transmission path of the vibrations at each meshing point to the sensor. Moreover, when failures occur in some tooth of the ring, sun, or planet, additional sidebands appear around the gear mesh frequency at integer multiples of the characteristic frequencies of each failure. Therefore, in the vibration signal of a PG the following components are differentiated:

1 High frequency component at gear mesh frequency ($f_{m}=Z_{r}f_{c}$). In this paper the value of $f_{m}$ is 432 Hz (see [22]).
2 Low frequency components corresponding to the transmission path envelope ($f_{A\left(t\right)}=N_{p}f_{c}$) or faults at ring ($f_{fr}=N_{p}f_{c}$), sun ($f_{fs}=N_{p}\frac{Z_{r}}{Z_{s}}f_{c}$), and planet ($f_{fp}=\frac{Z_{r}}{Z_{p}}f_{c}$) gears.

Therefore, Equation (7) can be interpreted as a band pass signal centered around frequency $w_{m}=2\pi f_{m}$, where the side bands contain the information that is required to diagnose the correct operation of the PG; thus, this signal can be represented using its low pass equivalent form, namely, in-phase (I) and quadrature (Q) components, following the canonical expression:

(8) $$v\left(t\right)=v_{I}\left(t\right)\cos\left(w_{m}t\right)-v_{Q}\left(t\right)\sin\left(w_{m}t\right)$$

Manipulating (7) based on (8) and using $A_{ri}\left(t\right)$ to refer to the modulation due to the transmission path for simplicity, the vibration signal can be represented as

(9) $$\begin{array}{c} v\left(t\right)=cos\left(w_{m}t\right)\left[V_{rp}+V_{sp}\right]\overset{N_{p}}{\underset{i=1}{\sum }}\left[A_{ri}\left(t\right)\cos\left(\frac{w_{m}\Psi_{i}}{w_{c}}\right)+kA_{ri}\left(t\right)\cos\left(\frac{w_{m}\Psi_{i}}{w_{s}-w_{c}}\right)\right]\\ -\sin\left(w_{m}t\right)\left[V_{rp}+V_{sp}\right]\overset{N_{p}}{\underset{i=1}{\sum }}\left[A_{ri}\left(t\right)\sin\left(\frac{w_{m}\Psi_{i}}{w_{c}}\right)+kA_{ri}\left(t\right)\sin\left(\frac{w_{m}\Psi_{i}}{w_{s}-w_{c}}\right)\right] \end{array}$$

Comparing (9) with (8) the I and Q components are:

(10) $$v_{I}\left(t\right)=\left[V_{rp}+V_{sp}\right]\overset{N_{p}}{\underset{i=1}{\sum }}\left[A_{ri}\left(t\right)\cos\left(\frac{w_{m}\Psi_{i}}{w_{c}}\right)+kA_{ri}\left(t\right)\cos\left(\frac{w_{m}\Psi_{i}}{w_{s}-w_{c}}\right)\right]$$

(11) $$v_{Q}\left(t\right)=\left[V_{rp}+V_{sp}\right]\overset{N_{p}}{\underset{i=1}{\sum }}\left[A_{ri}\left(t\right)\sin\left(\frac{w_{m}\Psi_{i}}{w_{c}}\right)+kA_{ri}\left(t\right)\sin\left(\frac{w_{m}\Psi_{i}}{w_{s}-w_{c}}\right)\right]$$

Note that knowing $v_{I}\left(t\right)$, $v_{Q}\left(t\right)$, and $w_{m}$, the function v(t) is completely defined. Thus, we can use this representation to elaborate the proposed method. A practical approach to obtain the low pass components of measured signals is multiplying (9) by $2\cos\left(w_{m}t\right)$ and $-2\sin\left(w_{m}t\right)$, followed by a low pass filter, respectively.

**Figure 3:**
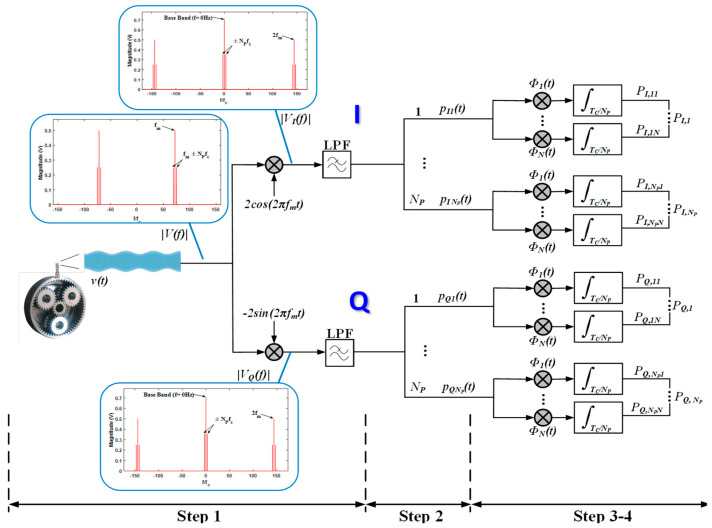
Block diagram of the process to obtain the geometric representation of the vibration signal of a PG.

The detailed processing scheme used to analyze the vibration signal of a PG is shown in Figure 3. First, the baseband in-phase (I) and quadrature (Q) components are obtained from the vibration signal detected by the sensor (using appropriate band pass filtering; in this case, around $f_{m}\pm 40f_{c}$). To achieve this, it is necessary to multiply the signal by a cosine and a sine at gear mesh frequency and subsequently low pass filter the resulting signals. The cutoff frequency of the low pass filter must be set so that the low frequency components of $v_{I}\left(t\right)$ and $v_{Q}\left(t\right)$ are preserved and the double frequency component (2$f_{m}$), is rejected. For the experimental case described in this paper, the cutoff frequency of the Butterworth low pass filter employed is $f_{m}$/2. From there, the $T_{C}$ period of each baseband component is divided in $N_{P}$ sections of duration $T_{C}/N_{P}$ corresponding to each planet $p_{Ii}\left(t\right)$ and $p_{Qi}\left(t\right)$ in Figure 3. Each of these sections is projected on N time basis functions, represented in the diagram as $\emptyset_{j}$, with j=1,2,…,N, through a multiplication and integration over its duration $T_{C}/N_{P}$. In this proposal, the basis will be obtained using the Gram–Schmidt method on a healthy PG. At the end of this process, $2\left(N\times N_{P}\right)$ scalars $P_{I,ij}$ and $P_{Q,ij}$ are obtained, with $i=1,2,\ldots ,N_{P}$ the planet index, with $N_{P}$ = 3 in the PG analyzed in this paper, and j=1,2,…,N the index of the basis functions.

As mentioned before, in this case, the number of basis obtained is *N* = *N_P_* = 3. These scalars can be graphically represented with the basis functions as the axes, obtaining points in a space known as a constellation [23]. This way, with the scheme of Figure 3, the signal under analysis can be compared with the signal of a healthy PG graphically, enabling faults to be detected by means of changes with respect to the “healthy constellation”. This is possible because the added frequency components due to faults displace the points in the constellation from their ideal positions because of phase and/or magnitude variations. The error or Euclidean distance between points of a healthy and faulty constellation can be analyzed as well to quantify the variation.

One of the main advantages of this method, compared with other methods used in the industry, is that it takes not only the magnitude into account, but also the phase of the vibration signal. In fact, the in-phase (I) and quadrature (Q) components of a signal can be interpreted with module $\sqrt{I^{2}+Q^{2}}$ and phase $tan^{-1}\left(Q/I\right)$ of (7). Due to this, there is an extra source of information in the analysis of the vibration signal and detection of faults.

Next, the process depicted in Figure 3 will be explained systematically, based on the model described above for a vibration signal of a healthy PG.

Step 1: Multiplication of the signal in (10) by $2\cos\left(2\pi f_{m}\right)$ and $-2\sin\left(2\pi f_{m}\right)$ and lowpass filtering to obtain the baseband I and Q components.

After the multiplication of the vibration signal v(t) by $2\cos\left(2\pi f_{m}\right)$ and −$2\sin\left(2\pi f_{m}\right)$, the frequency component at gear mesh frequency is moved to baseband and replicas of said component appear at double gear mesh frequency. The resulting spectra have been added to Figure 3 for a generic case. However, the absence or existence of a Q baseband magnitude component will depend on whether the gear mesh processes are in phase or not, respectively. Thus, it will depend on the group that PG belongs to, as described above.

**Figure 4:**
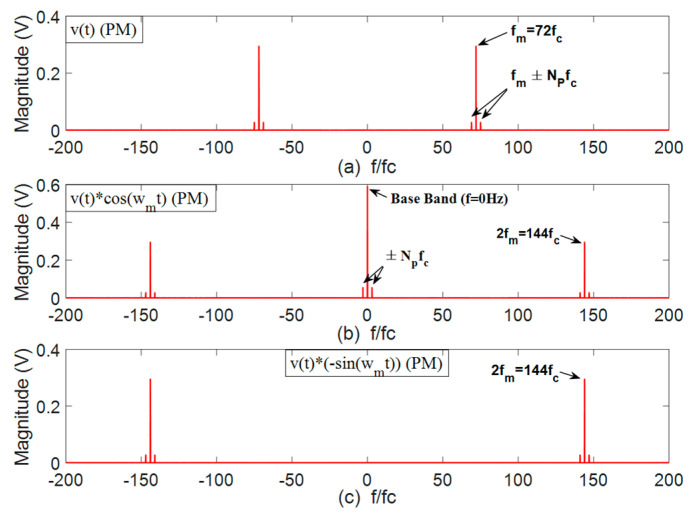
Spectra of the in-phase and quadrature component extraction process in Step 1. (**a**) Signal *v(t)* (**b**) Signal *v(t)·cos(w_m_t)* (**c**) Signal *-v(t)·sin(w_m_t)*.

In order to retrieve the information of $\;v_{I}\left(t\right)$, and $v_{Q}\left(t\right)$, components at double gear mesh frequency must be filtered out by using a low pass filter with the adequate cut-off frequency. Analyzing (10) and (11), it can be seen that baseband I and Q components are composed of the transmission path amplitude modulation function multiplied by the summation of cosine or sine terms of the angular position of each planet, for both planet–ring and planet–sun mesh processes. However, when there is a fault in a gear, the added frequency components are expected to alter (10) and (11) and, therefore, the resulting constellation. In Figure 4a the spectrum of v(t) is shown for the particular PG analyzed in this paper, which belongs to group A. Figure 4b shows the spectrum of $v_{I}\left(t\right)$ as described in (10) and Figure 4c the spectrum of $v_{Q}\left(t\right)$ as described in (11). Note that the resulting components of $v_{I}\left(t\right)$ are in phase and, therefore, are added; on the other hand, the components of $v_{Q}\left(t\right)$ are in counterphase, so they nullify each other. Thus, ideally, the baseband quadrature component of the vibration signal will be null for PG’s whose gear mesh processes are in phase.

Step 2: Division of $v_{I}\left(t\right)$ and $v_{Q}\left(t\right)$ in $N_{P}$ sections of duration $T_{C}/N_{P}$ each. The set of functions generated in this step from the vibration signal of a healthy PG will be used to obtain the basis (set of basis functions).

Step 3: Calculation of the basis functions of the vibration signal v(t) using the Gram–Schmidt method [23], which employs the set of signals obtained in Step 2 from a healthy PG. The main theoretical concepts of this representation technique are summarized below.

The geometric representation of the signals is based on representing any set of $N_{P}$ transmitted signals $p_{i}\left(t\right)$ as a linear combination of N orthonormal basis functions where $N\leq N_{P}$. That is, given a set of transmitted signals,$\;p_{1}\left(t\right)$, $p_{2}\left(t\right)$, …, $p_{N_{P}}\left(t\right)$, each of them of duration $T=T_{C}/N_{P}$, we define:

(12) $$p_{i}\left(t\right)=\overset{N}{\underset{j=1}{\sum }}p_{ij}\emptyset_{j}\left(t\right)dt,\;\;\{\begin{array}{c} 0\leq t\leq T\\ i=1,\;2,\;\ldots ,\;N_{P} \end{array}$$

where the coefficients of the expansion are described as follows:

(13) $$p_{ij}=\int_{0}^{T}p_{i}\left(t\right)\emptyset_{j}\left(t\right)dt,\;\;\{\begin{array}{c} i=1,\;2,\;\ldots ,N_{P}\\ j=1,\;2,\;\ldots ,\;N \end{array}$$

The real-valued basis functions $\emptyset_{1}\left(t\right)$, $\emptyset_{2}\left(t\right)$, …, $\emptyset_{N}\left(t\right)$ form an orthonormal set, therefore fulfilling the following conditions:

(14) $$\int_{0}^{T}\emptyset_{i}\left(t\right)\emptyset_{j}\left(t\right)dt=\{\begin{array}{c} 1\;if\;i=j\\ 0\;if\;i\neq j \end{array}$$

The first condition of the above formula defines that each basis function is normalized. The second condition means that the basis functions $\emptyset_{1}\left(t\right)$, $\emptyset_{2}\left(t\right)$, …, $\emptyset_{N}\left(t\right)$ are orthogonal to each other along the interval 0≤t≤T. The set of coefficients ${\left\{p_{ij}\right\}}_{j=1}^{N}$ can be seen as a signal vector of N dimensions, known as $p_{i}$.

**Figure 5:**
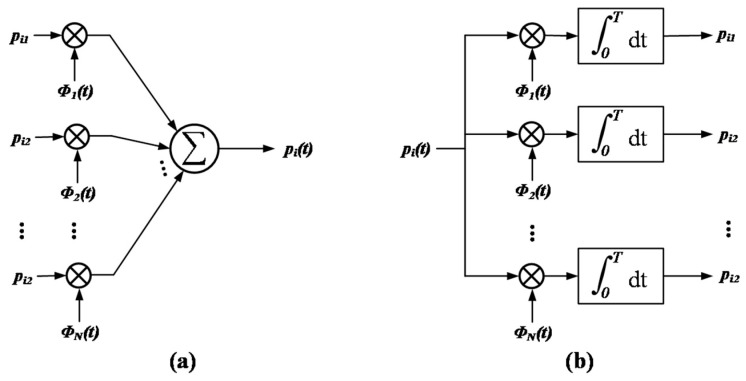
(**a**) Synthesizer for generating the signal *p_i_(t)*. (**b**) Analyzer for reconstructing the signal.

- Given the N elements of the vector $p_{i}$ as input, the scheme of Figure 5a can be used to generate the signal $p_{i}\left(t\right)$.
- In the same way, given the signal $p_{i}\left(t\right)$, $i=1,2,\ldots ,N_{P}$ as input, the coefficients $p_{i1}$, $p_{i2}$, …, $p_{iN}$ can be calculated following the scheme of Figure 5b whose geometric representation is known as constellation.

**Figure 6:**
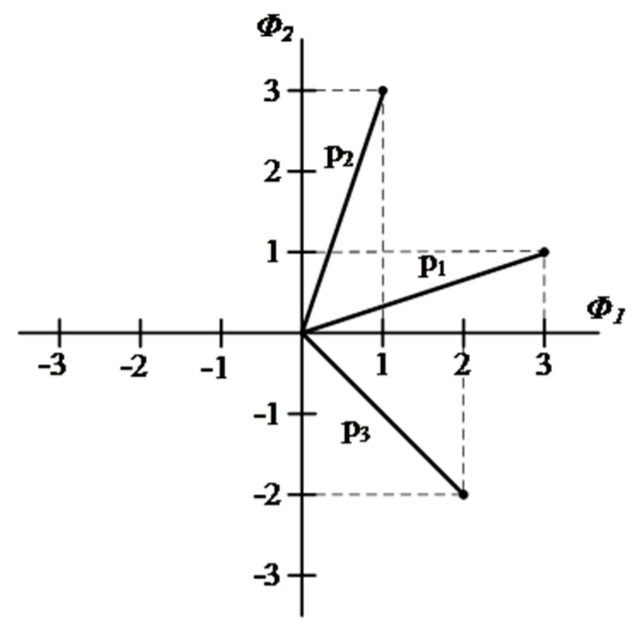
Geometric representation of signals with *N* = 2 and *N_P_* = 3 [23].

In this way, it can be stated that each signal in the set $\left\{p_{i}\left(t\right)\right\}$ is completely determined by the signal vector $p_{i}={\left[p_{i1},p_{i2},\cdots ,p_{iN}\right]}^{T},\;with\;i=1,2,\;\ldots ,\;N_{P}$. The set of vectors $\left\{p_{i}|\;i=1,\;2,\;\ldots ,\;N_{P}\right\}$ can be visualized as the definition of the corresponding set of $N_{P}$ points in an Euclidean space of N dimensions, with N axes perpendicular to each other named $\emptyset_{1}\left(t\right)$, $\emptyset_{2}\left(t\right)$, …, $\emptyset_{N}\left(t\right)$. This Euclidean space of N dimensions is known as signal space. Figure 6 shows an illustration of the geometric representation of signals, also known as a constellation, in a two-dimensional signal space (N = 2) with 3 signals ($N_{P}$ = 3).

In order to find $\emptyset_{i}\left(t\right)$, the following procedure is employed:

(15) $$\emptyset_{1}\left(t\right)=\frac{p_{1}\left(t\right)}{\sqrt{\int_{0}^{T}p_{1}^{2}\left(t\right)dt}}\;\begin{array}{c} g_{i}\left(t\right)=p_{i}\left(t\right)-\overset{i-1}{\underset{j=1}{\sum }}p_{ij}\emptyset_{j}\left(t\right)dt,\;\\ \emptyset_{i}\left(t\right)=\frac{g_{1}\left(t\right)}{\sqrt{\int_{0}^{T}g_{1}^{2}\left(t\right)dt}} \end{array}\;\;\;\;\;\;for\;i=2,\;3,\;\ldots ,\;N$$

In the experimental case exposed in this paper, the number of resulting basis functions orthogonal to each other is N = 3, rendering a three-dimensional signal space. In a constellation, the Euclidean distance between points is a very useful metric, especially when they are modified for external factors due to gears fault.

The Euclidean distance $d_{ik}$ between the vector representations $p_{i}\left(t\right)$ and $p_{k}\left(t\right)$ can be defined by:

(16) $$d_{ik}=||p_{i}-p_{k}||{}^{2}=\overset{N}{\underset{j=1}{\sum }}{\left(p_{ij}-p_{kj}\right)}^{2}=\int_{0}^{T}{\left(p_{i}\left(t\right)-p_{k}\left(t\right)\right)}^{2}dt$$

This magnitude can be used to detect faults on a PG. If $p_{i}\left(t\right)$ is the healthy signal and $p_{k}\left(t\right)$ is the measured one, then $d_{ik}\approx 0$ for a correct PG.

Step 4: Multiplication of the baseband I and Q components ($p_{Ii}\left(t\right)$ and $p_{Qi}\left(t\right)$ (respectively) by their basis function and integration over the period of each planet. Thus, with 3 planets, for planet 1 the integral will be performed over 0 to $T_{c}/N_{P}$ section, for planet 2 over $T_{c}/N_{P}$ to $2T_{c}/N_{P}$, and for planet 3 over $2T_{c}/N_{P}$ to $T_{c}$. This way, $N_{P}$ vectors associated to each planet are obtained, which can be represented in a constellation in the signal space diagram.

Following these steps using the vibration signal of a healthy PG, an ideal constellation is obtained, which can be used as a reference in comparison with the constellation obtained for a PG under analysis in order to detect faults. In the next section, some experimental results are shown to demonstrate the proposed technique.

## 3. Results

The experimental measurements are taken in the test rig described in [22] as TR2, which includes a single-stage spur-gear planetary gearbox, an asynchronous motor, a data acquisition system, and a magnetic brake controlled by a current signal as the loading unit.

Three different vibration signals were analyzed for the conceptual validation of the proposed fault detection method: the vibration signal obtained from a healthy PG (to be used as a reference), the vibration signal of a PG with a fault in the ring gear, and the vibration signal of a PG with a fault in the sun gear.

**Figure 7:**
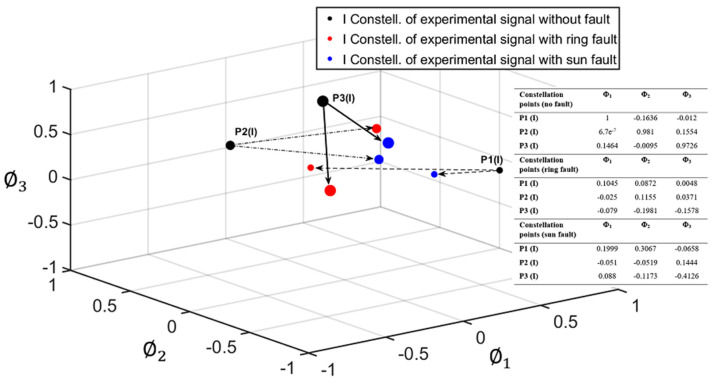
In-phase (I) constellation of a healthy PG (black), PG with a fault in the ring gear (red), and PG with a fault in the sun gear (blue).

**Figure 8:**
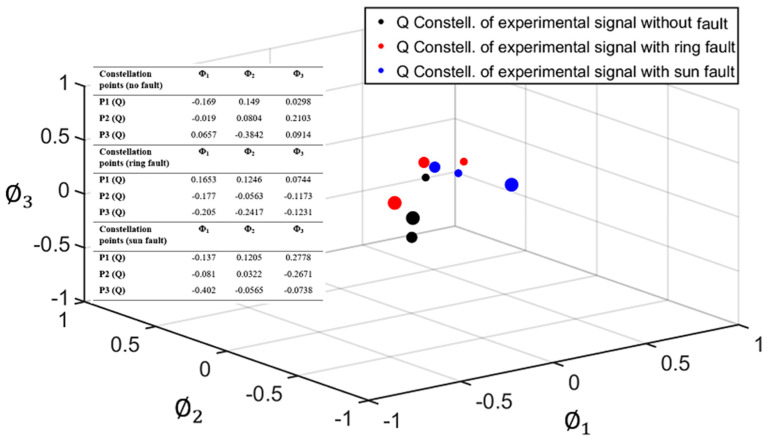
Quadrature (Q) constellation of a healthy PG (black), PG with a fault in the ring gear (red), and PG with a fault in the sun gear (blue).

As explained in Section 2, for the geometric representation of each signal under study, two separate constellations were obtained, corresponding to the I and Q components of each signal (see Figure 3). Each constellation is composed of points (vectors of $N_{P}$ dimensions) representing each planet. Figure 7 and Figure 8 show the I and Q constellations of the vibration signals of a healthy PG (black), a PG with a fault in the ring (red), and a PG with a fault in the sun (blue) in the same signal space, using the total duration of the signal and, therefore, obtaining one point per planet. It can be observed in Figure 7 that when a fault exists, the points move from their healthy positions. These movements have been represented with a dashed arrow for planet one, dash-point arrows for planet two, and thick arrows for planet three. Note that, in this case, gear-meshing processes are in-phase [19] so that the Q component is expected to be almost zero as can be seen in Figure 7 where the points are distributed near zero in all three axes. Thus, for the cases in which the gear-meshing processes are in-phase, the Q constellation will not be representative to detect faults in the PG.

As additional numerical information for fault detection, the Euclidean distance between points in the “faulty constellations” and their equivalent points in the “healthy constellations” were obtained (see Table 1).

Since the vibration signals are susceptible to changes due to external interferences, noise, PG state variations, etc., that change over time, the signals have been analyzed over various cycles obtaining a constellation for each cycle.

**Figure 9:**
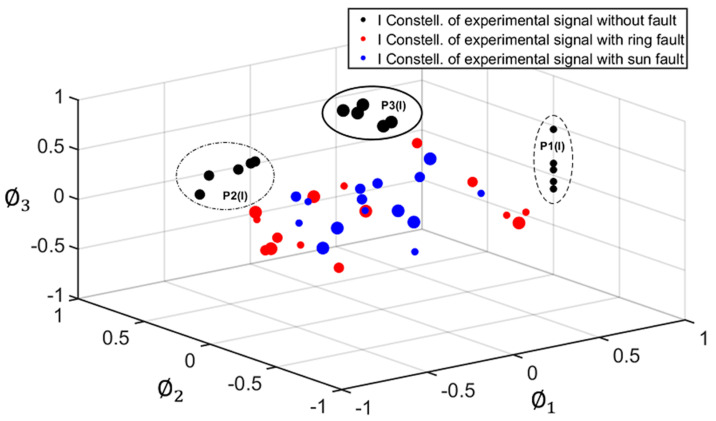
In-phase (I) constellation of a healthy PG (black), PG with a fault in the ring gear (red), and PG with a fault in the sun gear (blue). Representation of clouds of points.

**Figure 10:**
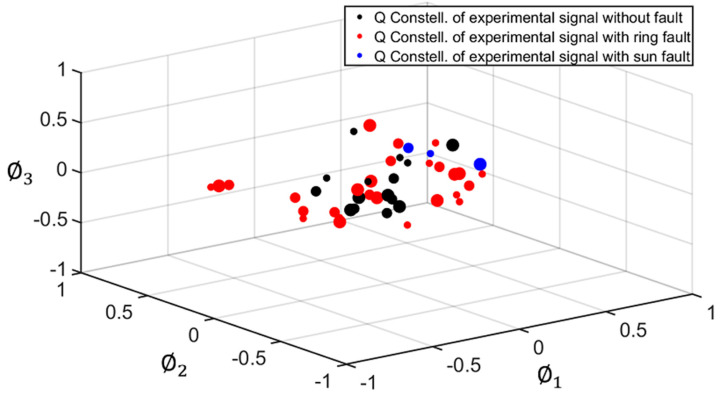
Quadrature (Q) constellation of a healthy PG (black), PG with a fault in the ring gear (red), and PG with a fault in the sun gear (blue). Representation of clouds of points.

This way, each planet is defined by a cloud of points instead of a single point. In Figure 9, these clouds are delimited by a dashed-line circle for planet one, a dash-point line circle for planet two, and a thick line circle for planet three. The shifts of points inside the clouds in the case of a healthy PG will be due to the changes mentioned before, while larger shifts, resulting in points outside the clouds, will be caused by faults in the ring or the sun gears. Figure 9 and Figure 10 show the I and Q constellations obtained for this analysis, corresponding to five cycles of each signal. As expected, in Figure 9 it can be observed that the points of each planet form a compact cloud when there is no fault in the PG under analysis. However, when there is a fault in the ring or sun gear, this can be detected as the points of the resulting constellations fall outside of the clouds.

## 4. Discussion

A novel vibration based PG fault detection method is proposed, which provides additional information about the behavior of, not only the magnitude, but also the phase of the vibration signal detected by the sensor depending on the state of the PG.

To validate the method, experimental PG vibration signals under different faulty conditions were analyzed. Comparing experimental vibration signal on healthy and faulty conditions, the displacement of points in the constellation and the resulting Euclidean error values indicate the existence of faults.

Condition monitoring of PG’s for fault detection is currently a prominent field of research. The vibration signal processing method developed in this paper is a versatile method that can contribute to said field, since it provides suitable fault indicators for different needs. On the one hand, the constellation of the baseband I and Q components of the vibration signal allows observing the displacements of the points with respect to their ideal positions. This permits an online monitoring of the status of the PG throughout its operation. On the other hand, the error or Euclidean distance between the constellation points of the analyzed PG and their ideal positions provides a quantitative assessment of the status of the PG.

Note that due to the filtering carried out to extract the I and Q components (see Figure 3), out-of-band noise is rejected. Hence only in-band noise is relevant. Due to the sideband features, the required filter bandwidth and hence the influence of noise on the signal relies on the particular PG, specifically on the number of planets and the carrier rotation frequency. Besides, the scheme of Figure 3 relies on correlators to process the I and Q signals, which is a rather robust technique against noise in the same manner as correlation receivers in communications [23].

Future research directions include the extension to progressive damage detection and nonstationary vibration signals. Progressive damage [24,25,26] of the PG could be potentially detected using the proposed technique by monitoring the evolution with time of the Euclidean distance between the points of the constellation that correspond to a healthy PG and those of the PG that are experiencing progressive damage. This idea assumes that such progressive damage is causing a variation on the I and Q signal components expressed in Equations (10) and (11). This assumption is reasonable since the progressive pitting on the gear tooth surface leads to an increase in the vibration [24].

Concerning nonstationary vibration signals, they may arise from variations in the rotation speed. In this case, a technique should be included in Figure 3 that allows tracking phase and frequency variations of the carrier in the generation of the tones applied to the input multipliers. The situation is similar to that of a communication receiver based on synchronous detection. For this purpose, a phase locked loop (PLL) can be used in Figure 3 that would be in charge of generating the synchronous signals 2cos(2π*f_m_*t) and −2sin(2π*f_m_*t) from the vibration signal *v(t)*.

Finally, the inclusion of data-driven techniques can also be explored [27].

## 5. Patents

A Spanish patent was submitted by the authors on 28 September 2018 (number P201830943) protecting the technique described in this paper.

## Figures and Tables

**Table 1:** Normalized Euclidean distance to constellation of a healthy PG and a faulty PG.

Fault Location	I Constellation	Q Constellation
Ring gear	d_P1_ = 1.53	d_P1_ = 0.211
	d_P2_ = 1	d_P2_ = 0.69
	d_P3_ = 1.2	d_P3_ = 0.148
Sun gear	d_P1_ = 0.733	d_P1_ = 0.14
	d_P2_ = 1.066	d_P2_ = 0.72
	d_P3_ = 0.779	d_P3_ = 0.51

## Data Availability

Not applicable.
